# Bleuler's Psychopathological Perspective on Schizophrenia Delusions: Towards New Tools in Psychotherapy Treatment

**DOI:** 10.3389/fpsyt.2018.00306

**Published:** 2018-07-17

**Authors:** Filipe Arantes-Gonçalves, João Gama Marques, Diogo Telles-Correia

**Affiliations:** ^1^CliniPinel, Lisbon, Portugal; ^2^Clínica de Saúde Mental do Porto, Porto, Portugal; ^3^Clínica Universitária de Psiquiatria e Psicologia Médica, Faculdade de Medicina, Universidade de Lisboa, Lisbon, Portugal; ^4^Consulta de Esquizofrenia Resistente, Centro Hospitalar Psiquiátrico de Lisboa, Lisbon, Portugal; ^5^Serviço de Psiquiatria e Saúde Mental, Hospital de Santa Maria, Centro Hospitalar de Lisboa Norte, Lisbon, Portugal

**Keywords:** affectivity, Bleuler, delusions, schizophrenia, psychopathology

## Abstract

The authors begin by addressing the historical evolution of the delusion concept and its different approaches, focusing afterwards mainly on the work of Bleuler, who stressed the proximity between delusions and the emotional life of patients with schizophrenia. I Therefore, the present work intends to review the main aspects of the theory of delusion formation in schizophrenia according to Bleuler's psychopathological perspective. For that purpose, first the role of delusions in the psychopathology of schizophrenia is explored in a close relation with the Bleuler's fundamental symptoms (Alogia, Autism, Ambivalence, and Affect Blunting) nowadays known as negative symptoms. Then, persecutory, grandiosity and sexual delusions in schizophrenia are described according to the tension between logic and affects, as well as, internal conflict, schizoid features, and auto-erotism as key psychopathological pathways. Thus, with this subjective perspective, it is intended to highlight Bleuler's psychopathological contribution to the affective and meaningful causality of delusions in schizophrenia. The former might be useful in the integration with other psychopathological phenomena (hallucinations and negative symptoms) and new forms of research and therapeutic approaches in this disorder that are complementary with the contemporary tendencies in psychopathology.

## Introduction

Throughout the history of psychopathology the term delusion had several meanings distant from its current meaning of thought disorder (1). In antiquity and in the eighteenth and nineteenth centuries' French Psychiatry, the term *délire* (delusion), meant general detachment from reality that was not specific of thought impairment (1).

In eighteenth and nineteenth centuries' French Psychiatry, the term delusion included disturbances of thought, perception, emotions and affects and even psychomotricity (2). By contrast, in the twentieth century German and British Psychiatry, the term delusion gradually became synonymous with a false belief, a disorder of the thought content (3). This tendency was generalized in the majority of the European countries and also in the United States of America, with the replacement of the old broader concept by a newer and narrower concept of delusion as a disorder of thought content.

Jaspers defined delusion as a disorder in the content of thought, separating it from other psychopathological disorders such as perception, affect and personality's. He considered primary delusion incomprehensible and in discontinuity with the individual's personality (4).

However, throughout the twentieth century, some authors such as Freud and Bleuler were not satisfied with the Jasperian definition of primary delusion as an isolated and incomprehensible phenomenon, and tried to integrate it in the general psychic life of patients.

For Freud, delusions result from a conflict between the ego and the external world that makes the former lose its contact with reality, mainly because of an intolerable frustration (5). He assumes that delusions might occupy the place left by that loss of contact with reality (6). These efforts to recapture the outside world through delusion occur in continuity with the emotional memories of the patient, previous to reality detachment (5).

Bleuler thought much about delusion in schizophrenia, namely its relation with affect, personality and the proximity with what he called the fundamental symptoms (nowadays known as negative symptoms of schizophrenia). Bleuler was the first author to gather descriptive and analytical perspectives on the psychopathology of schizophrenia. He added comprehensive and interpretative components without forgetting the importance of psychopathological description and systematization. This is an example of how it is possible to integrate different paradigms regarding the same psychopathological phenomenon.

In this article, we intend to review the main aspects related to the theory of the formation of delusion in schizophrenia, according to Bleuler's psychopathological perspective.

## The role of delusions in schizophrenia psychopathology

Bleuler systematizes the clinical presentation of schizophrenias into fundamental, accessory, primary and secondary symptoms. The fundamental symptoms, which are virtually present through all the course of the disorder (7), are also known as the famous Bleuler's four A's: Alogia, Autism, Ambivalence, and Affect blunting (8). Delusion is regarded as one of the accessory symptoms because it is episodic in the course of schizophrenia. Among the primary symptoms one can find alogia that Bleuler claimed to have a neurological etiology. All the remaining symptoms, including delusion, are considered secondary symptoms because they are an attempt of psychogenic compensation of the deficits caused by alogia. Bleuler conceptualized delusion as an accessory and secondary symptom in schizophrenia's psychopathology in very close relation with fundamental symptoms.

In alogia, as the logical thought weakens, affects become predominant and dominate the associations of the thinking processes (9). Based on this hypothesis, Bleuler described a link from alogia to delusion formation, with wishes and fears dominating the association of thoughts, bringing way to autistic thought, withdrawing the patient from external reality, predisposing him to delusion formation (7).

Regarding autism, it can be conceptualized as the predominance of inner life that distances the patient from external reality. In this sense, autism can be seen as a difficulty in contact with others (auto-erotism) but also as social isolation and negativism predisposing the patient to delusion formation (10).

Concerning affect blunting, Bleuler argues that although affects seem to be decreased at superficial psychiatric observation, they are very intense at deeper layers of the psychic life of the patient (11). Affect blunting might predispose patients with schizophrenia to delusion specifically when interpersonal conflicts bring to surface those apparently hidden emotions.

Finally, ambivalence is described as a tendency to be in the presence of contradictory feelings. Bleuler described this ambivalence as much more intense, regarding anxiety, than the one present in neurotic (not psychotic) patients (12). Considering this intense anxiety in schizophrenia's ambivalence, delusions represent a psychopathological way of dealing with these internal and emotional conflicts.

## Theory of delusion formation in schizophrenia

According to Kraepelin (13), delusions were incorrect ideas created, not by an accidental failure of logic, but by an inner need of the patient (13). And for Bleuler and Brill (9) the most important inner needs are the affective ones. In that sense, delusions always follow a definite direction corresponding to the patients affects, and in the vast majority of cases cannot be corrected by new experience or instructions, as long as the condition which gave origin to them remains (9). Thus, delusions have their origin mainly in belief instead of logic. From Bleuler's point of view, delusions are frequently egocentric and very significant for the personality of the patient (7). By other words we can stress that the delusions thematic is mainly anchored in the patient's biography.

Bleuler acknowledges that the strength of affects (in affect blunting) combined with the weakness of logic (alogia) is the most common feature in delusions formation. When affects are present and strong, patients are more prone to logic errors, which mean that affects have a key-role in the formation of delusions (7). The latter ones might be conceptualized as stemming from unconscious thinking derived from the wide splitting of mental functions (9) where the autonomy of traumatic emotional memories becomes predominant. These traumatic emotional memories belong to the autistic way of thinking, based on the fantasies that are detached from reality. So autistic thinking and affective needs take advantage, over realistic and logical thinking, and patients become vulnerable to delusion formation (10).

## Psychopathological mechanisms in delusion formation in schizophrenia

As previously adressed, Bleuler argued that delusions were a secondary, psychogenic, kind of symptom, involving different specific psychopathological mechanisms: internal conflict, schizoid features, and auto-erotism (12).

In internal conflict we can assume that traumatic emotional memories have influence in realistic thinking, giving rise to conflict between internal and external reality. Moreover, there is a tension because of the imbalance between pleasant and unpleasant affects and delusion formation is the only way allowing traumatic emotional memories to manifest (11).

Regarding schizoid features, Bleuler claimed that these personality traits are essential and in accordance with the autistic way of thinking (11). This kind of thinking, based on fantasies turns the patient away from reality, liberating subjective wishes, but without further adaptation (10). It always seeks pleasure and avoids pain. Freud argued that schizophrenic delusions are not only wish-fulfilling but also the attempt to recapture lost internal objects (6).

Finally, auto-erotism is as a key-feature of autism in schizophrenia as negativism has frequently an erotic side that may be pleasant as flirting, unpleasant as harassment, or both at the same time (12).

## Persecutory delusions

In this kind of delusion, Bleuler considers that there is frustration after a great ambition of the patient is not achieved (11). The patient is kept in an internal conflict between denying and accepting this frustration that may decrease his self-esteem, damaging his narcissism. Many patients in this situation cannot deal with failure and project their guilt feelings in people around them (7). Without this contradiction between ambition (wishes) and reality (possibilities) there would be no delusion of persecution (11). In other words, first patients don't have what they wish, then they don't admit their incompetency and the result is the delusion of persecution, blaming others for their failure (9). Delusion of persecution is the most common type of delusion in schizophrenia (7).

## Grandiosity delusions

Very frequently grandiosity delusion is secondary to persecutory delusion (6, 14, 15). When the projection of guilt (persecutory delusion) fails to balance internal wishes and external reality, delusion of grandiosity may occur as a fulfillment of the repressed wish (11). As the external reality contradicts the guilt projected into the outside world, narcissistic injury to self-esteem grows, leaving the patient with the escape of wish-fulfillment through delusion of grandiosity (7). In other words the patient justifies his persecution delusion with a grandiose explanation, feeling him as an extremely important person, thus restoring his fragile self-esteem (9). Delusion of grandiosity is the second most common type of delusions in schizophrenia (7).

## Sexual delusions

This kind of delusions is also very common in schizophrenia. Usually, the patient believes it is forbidden for him to do what he wishes, under threats of danger, or punishment (12). Bleuler conceptualized that sexual thematic memories have a prominent role in schizophrenia as many patients presented sexual delusions of being loved (delusional erotomania), abused (delusional rape), or pregnant (delusional gestation). According to Bleuler, sexual delusions are a combination of persecutory and grandiosity delusions (7) and can also express the traumatic emotional memories that belong to the autistic way of thinking.

## Discussion

Nowadays the biological paradigm has monopolized psychopathology's studies, leaving meaning and symbolic causalities behind. This approach brought a reductive and poor view of psychopathology which could and should be enriched with other lines of thought.

For Bleuler, patients' affects are extremely important in the formation of delusions in schizophrenia, and this perspective may be useful in the investigation of new forms of therapeutic approach of this disorder. It also represents humanistic and patient-centered approach regarding the patient with schizophrenia, and reflects what is actually observed in clinical practice.

Bleuler also pointed out that delusions cannot be evaluated and studied separately from the rest of psychopathology. This view is in agreement with several authors of French psychopathologists (e.g., Esquirol and Henry Ey) for whom delusions were very close to other psychopathological phenomena such as hallucinations, an interdependence that has already been approached conceptually and empirically by more recent authors (16–18).

Another important aspect of Bleuler's vision is the proximity between positive and negative symptoms. For Bleuler they are strongly linked, with the negative symptoms preceding the positive symptoms (e.g., delusions).

In sum, with Bleuler, schizophrenia deserves to be approached not only from an objective perspective but also from a subjective perspective (taking into account the affective component, and the symbolic causality) in order to grasp the real picture of what is happening inside the patients.

New research could be based on this line of thought. Namely the study of the role of psychological trauma and emotional memory in schizophrenia patients' psychopathology, trying to add complementary knowledge to genetic studies, building bridges between genetics and environment (nowadays called epigenetics); On the other hand it would be interesting to assess the effectiveness of psychotherapies (which focus on factors related to the affective and the meaningful components of symptoms), alone or in combination with psychopharmacology in the treatment of schizophrenia.

## Author contributions

FA-G conceptualized and wrote the first draft of the manuscript. JG contributed with commentaries and suggestions. DT-C reviewed and supervised all the writing process.

### Conflict of interest statement

The authors declare that the research was conducted in the absence of any commercial or financial relationships that could be construed as a potential conflict of interest.
